# The risks of partner violence following HIV status disclosure, and health service responses: narratives of women attending reproductive health services in Kenya

**DOI:** 10.7448/IAS.19.1.20766

**Published:** 2016-03-31

**Authors:** Manuela Colombini, Courtney James, Charity Ndwiga, Integra team, Susannah H Mayhew

**Affiliations:** 1 Department of Global Health and Development, London School of Hygiene and Tropical Medicine, London, UK; 2 Youth Programme, Save the Children US, London, UK; 3 Reproductive Health Program, Population Council, Nairobi, Kenya

**Keywords:** HIV-positive women, women living with HIV, intimate partner violence, violence against women, HIV disclosure

## Abstract

**Introduction:**

For many women living with HIV (WLWH), the disclosure of positive status can lead to either an extension of former violence or new conflict specifically associated with HIV status disclosure. This study aims to explore the following about WLWH: 1. the women's experiences of intimate partner violence (IPV) risks following disclosure to their partners; 2. an analysis of the women's views on the role of health providers in preventing and addressing IPV, especially following HIV disclosure.

**Methods:**

Thirty qualitative interviews were conducted with purposively selected WLWH attending clinics in Kenya. Data were coded using NVivo 9 and analyzed thematically.

**Results:**

Nearly one third of the respondents reported experiencing physical and/or emotional violence inflicted by their partners following the sero-disclosure, suggesting that HIV status disclosure can be a period of heightened risk for partner stigma and abuse, and financial withdrawal, and thus should be handled with caution. Sero-concordance was protective for emotional and verbal abuse once the partner knew his positive status, or knew the woman knew his status. Our results show acceptance of the role of the health services in helping prevent and reduce anticipated fear of partner stigma and violence as barriers to HIV disclosure. Some of the approaches suggested by our respondents included couple counselling, separate counselling sessions for men, and facilitated disclosure. The women's narratives illustrate the importance of integrating discussions on risks for partner violence and fear of disclosure into HIV counselling and testing, helping women develop communication skills in how to disclose their status, and reducing fear about marital separation and break-up. Women in our study also confirmed the key role of preventive health services in reducing blame for HIV transmission and raising awareness on HIV as a chronic disease. However, several women reported receiving no counselling on safe disclosure of HIV status.

**Conclusion:**

Integration of partner violence identification and care into sexual, reproductive and HIV services for WLWH could be a way forward. The health sector can play a preventive role by sensitizing providers to the potential risks for partner violence following disclosure and ensuring that the women's decision to disclose is fully informed and voluntary.

## Introduction

Intimate partner violence (IPV) is a global public health and a human rights issue with several negative health outcomes [1–4]. In low-income settings, the IPV risk among women living with HIV (WLWH) is consistently higher than in HIV-negative counterparts [5–8].

Though relatively small, there is some significant literature exploring HIV as a risk factor for IPV, reporting adverse outcomes including threats of violence; physical, verbal, and emotional violence; and separation and loss of financial support [9–12]. In particular, despite its potential preventive health benefits and care implications [13,14], HIV disclosure to sexual partners has been widely reported as a major source of stress for WLWH, with many fearing male partner stigma, discrimination, violence or abandonment [11,15–19]. Several are less likely to enrol in HIV care as a consequence of such stressors [20,21]. The literature is mixed, with one review of outcomes to HIV disclosure among antenatal care (ANC) women emphasizing the relatively low prevalence of actual negative consequences of HIV disclosure for women [13]. Nevertheless, for many WLWH, the disclosure of HIV-positive status can lead to either an extension of former violence or new conflict specifically associated with the HIV test [22].

Most of the literature on IPV and partner disclosure comes from studies among pregnant women at ANC and prevention of mother-to-child-transition (PMTCT) services [5,10–12,21,23–26]. A study among ANC clients in Nigeria found that for 74% of the women who reported abuse by a partner, the violence started after HIV diagnosis [11]. Other similar studies among pregnant women in South Africa [10] and Zimbabwe [12] also reveal that HIV partner disclosure can be a trigger for feared violence or relationship conflicts, even irrespective of their HIV status [12,26].

With international guidelines and protocols recommending HIV status disclosure to partners and couples testing and counselling as an important HIV prevention measure, more women are under pressure by health services, especially those dedicated to sexual and reproductive health (SRH), to bring their partners for testing [10]. Several funded and ongoing trials are testing models to “bring men into ANC clinics.” However, the socially adverse outcomes of HIV disclosure to partners and, particularly, the risk of partner violence are reported to be often undiscussed or poorly approached by health providers [27], even though some providers acknowledge disclosure fears when women are in violent relationships [10,28].

There are limited studies in SRH settings outside ANC services exploring IPV risks and fear of partner abuse following HIV diagnosis [9,18,27]. This study tries to address this gap and aims to:

1. explore WLWH experiences of IPV risks following disclosure to their partners, and
2. analyze women's views on the role of SRH health providers in preventing and addressing IPV, especially following HIV disclosure.

## Methods

Thirty semi-structured qualitative interviews were conducted with a group of HIV-positive women. Respondents were purposively selected to ensure wide facility representation, from a quantitative cohort of 179 SRH care attendees at 16 public health facilities participating in the Integra Initiative in Central and Eastern Provinces. The study design for Integra research on integration of reproductive health (family planning and postnatal care) and HIV services in Kenya and Swaziland is detailed elsewhere [29]. Inclusion criteria were:

1. living in the facility catchment area,
2. being HIV positive,
3. attending SRH services (PNC or FP),
4. aged at least 15 years, and
5. willing to give informed consent for interview.

Some respondents declined consent (due to fatigue), could not be traced (relocated from the residence at the time of interview) or had died. Eight female trained local interviewers conducted face-to-face interviews in Kiswahili in February 2012. Written informed consent was obtained from all participants prior to the interviews. Interviews were conducted in private locations chosen by the interviewees, took approximately one hour to complete and covered client experiences with integrated SRH services. Although the focus of the interviews was not violence, questions on HIV-related challenges, potential risks following disclosure and any IPV experiences were included. All interviews were audio-recorded, transcribed and then translated into English. The transcripts were coded by two authors using NVivo 9.0 and analyzed thematically, using exploratory and inductive coding [30]. A broad definition of IPV was used, encompassing sexual, physical, economic and psychological abuse. A thematic matrix was developed for each case to document IPV occurrences, association of occurrences with HIV status (and if so, how) and other emerging factors. Subsequently, emerging themes and discrepancies across them were discussed with co-authors and local partners to ensure validity of the findings. A coding hierarchy was adjusted and refined during analysis, until overarching themes were identified. Finally, emerging links and pathways were identified. Reporting adheres to Consolidated criteria for reporting qualitative research (COREQ) [31].

### Ethical clearance

Ethical clearance was granted by the Kenya Medical Research Institute (KEMRI) Ethical Review Board (#113 and 114), the Ethics Review Committee of the London School of Hygiene & Tropical Medicine (LSHTM) (#5426) and the Population Council's Institutional Review Board (#443 and 444). The Integra Initiative is a registered trial: ClinicalTrials.gov Identifier: NCT01694862.

## Results

Thirty women of reproductive age were interviewed qualitatively, the majority [25] of which were married or with a partner. Over half of them [19] had three or more children. Nearly all [28] of them were on antiretrovirals and were using family planning. Nearly one third [8] experienced abuse following HIV disclosure to their partner.

### Women's experiences of IPV post-HIV disclosure

Nearly all women reported having disclosed to their current or former partners, despite some fear of negative consequences, including partner conflict, abandonment by a husband, withdrawal of financial support, and of the husband's refusal to use condoms (for preventing unwanted pregnancies).

Nearly one third of the women reported experiencing partner violence post-HIV sero-disclosure. Four experienced direct physical violence (one extreme), while the remaining described experiencing controlling behaviour and emotional abuse, including denial of communication, accusations of infidelity, blame for “bringing the virus”; abandonment following status disclosure; sexual coercion (refusal to use condoms); and withdrawal of material support. In many cases, these abusive behaviours were typically co-occurring. None of the women disclosed any personal experience of sexual violence perpetrated by their partners. Only two women seemed to have experienced partner violence or conflict prior to HIV disclosure, while the others did not mention prior information on partner conflicts or violence.

Quite commonly reported were blame for spreading the disease, separation (temporary and permanent) and the resulting loss of material support. These were fears and reality that many of the HIV positive women interviewed reported as common among HIV-positive women in their communities.

I told the partner I had long time ago and he left me. He deserted me. He told me that I am the one who went looking for it [HIV] there; while him, he just stayed there. He wanted to beat me and we separated […] He refused to provide for the food and told me to go and look for the food where I got HIV. [01, 2 children, on ARVs, separated]

R: There was no support, it was punishment and I don't like remembering. He knew about my HIV status and the child's and yet he did not support us. [02, 2 children, on ARVs, separated]

### Risk factors for IPV post-disclosure

Relational triggers of IPV were the main risk factors reported post-disclosure; these included sero-discordance, asymmetrical disclosure and requesting the partner to get tested.

HIV sero-discordance increased the risk of partner violence for some women.

When my husband discovered that I was positive and he was negative, he did not want to see me. I was the cause for the children to be positive, he abused me and quarrelled me until everybody knew. […] It was bad because we used to fight daily without any reason. [02, 2 children, on ARVs, separated]

Disclosure was asymmetrical and gendered in most cases, with most women testing by themselves (without the partner) during pregnancy, heightening the risk for IPV as this enabled male partners to place blame on women, and potentially justify physical violence and separation.

However, some of the women who experienced emotional IPV since disclosure to their partners said that the abuse stopped once the partner also learned his own positive status.

R: he said he doesn't know anything about that because he is not sick, and said that I am the one who came with it. […] Then we talked with him until he calmed down [abuse stopped]. I was told to bring him to CCC, he accepted and I brought him to start his clinic. [03, 2 children, on ARVs, married]

Same status couples that tested together or entered the relationship knowing they were HIV positive seemed to experience less negative reactions to disclosure.

R: We were tested together. […] [His reaction was] Not bad, he said those are God's plans, so when we were tested and we all came to be positive so there was nothing but to pray and take medicines […] I didn't feel pain because I was also positive and if he was negative he could have sent me away. [04, 3 children, on ARVs, married]

Over half of the partners of the women who reported violence refused testing, and requesting a partner to get tested was seen as a potential trigger for relationship conflict and possibly violence. In two cases, initial friction started when the woman requested the partner to go for HIV testing and eventually respondents experienced IPV and separation.

First he got sick, I told him we go for VCT but he refused. I suspect he knew he was HIV positive. When I insisted we take the test, it caused antagonism between us. [05, 1 child, on ARVs, separated]

### Women's narratives of health service responses

#### Experiences with counselling and advice on disclosure

From some of the accounts, the advice received on disclosure was quite broad. Many were told to disclose to someone close in order to be supported when sick, though some women initially still expressed fear of disclosing and suggesting the need for additional support.

I was told I need to tell mother, my family, so that we know how to live together […] you fear at the beginning, you ask yourself where do I start from …. [02, 2 children, on ARVs, in relationship]

Others were also told to wait until the partner would go and get tested.

The first time I was tested and was found to be sick. I was told not to tell him directly. So that he can test first. [1 child, on ARVs, separated]

Some were warned about potential stigma following disclosure, though no women who experienced abuse reported any mention of IPV risk following disclosure during their post-test counselling.

I was asked [by providers at ANC] to go and come with him. When I went and explained to him, he asked me why he was being called and I told him the doctor is calling him. The doctor told me to come back with him. He didn't explain why he was calling him. [06, 1 child, on ARVs, separated]

Only one person mentioned that time constraints prevented any discussion on potential dangers following disclosure during her counselling session.

There is no time … the provider has never told me that there are dangers when disclosing. [07, 2 children, on ARVs, separated]

#### Views on role of health providers in addressing IPV

During the interviews, women were also asked whether or not IPV was an issue that health providers could help with. Many believed that health providers can support women who experienced IPV, especially after HIV disclosure, and most suggested counselling (following disclosure of violence) in various forms: counselling and psychosocial support, either individually to the men or the women or jointly; or mediated disclosure (Table 1).

**Table 1:** Views on potential role of health providers in dealing with IPV

Counsel woman and ask her to come back with the husband	“He [the provider] can only ask you to come with the partner so that he can talk to you, he will tell you that if your partner makes noise, you keep quiet.” [08, 1 child, on ARVs, married]
Counsel the husband on how to stay well with the wife and to stop the violence	“They [providers] should counsel husband on how to stay with the wife, arrange seminars for them, to give them teachings on how to stay with their partners, to listen to how others are staying with their partners.” [06, 1 child, on ARVs, separated]
Counsel couples to reduce partner abuse and separation	“… when wife is tested at ANC, before she is referred to CCC [comprehensive care centre for HIV services] they should call the husband for testing and if he tested, we should all be taken to CCC, sit down with husband and wife and talk to them, the husband can agree with what the wife is told, if it's the condom, they can use it. […] they discuss the process if there is guidance and counselling.” [10, 3 children, on ARVs, married]
Refer the case to counsellors for home visits to counsel husband about partner violence	“A woman would disclose [partner violence] to the provider and then the provider would call a counsellor on site and then the counsellor will do home visit to talk to your husband.” [09, 4 children, on ARVs, married]
Can report violent men	“When a woman goes for treatment, doctors can take legal action and take them to court if they abuse or molest women.” [02, 2 children, on ARVs, in relationship]
Cannot really help: - Can only help medically - Should not interfere in domestic issues	“The provider will only treat you if you have been hurt. The provider can only document, there is nothing else he or she can do.” [10, 3 children, on ARVs, married] “I don't think they [providers] should come [to help] because those are domestic issues and should be handled by the two parties.” [12,1 child, not on ARVs, separated]

Counselling men was said to be the way forward to educate men, as they are at the source of the IPV problem. Some women further suggested that health providers should discuss relationship and communication skills with their partners in order to reduce IPV.

Because they [providers] can talk to men … they can counsel them on how to relate to their wives. If it is something like quarrelling, they are counselled to sit down and discuss. […] I think the only thing is to counsel them [men], I don't think there's anything else they can do because if you help a woman and it is the man who has a problem, what would you be helping? [13, 3 children, on ARVs, married]

However, several women were unsure of how much impact health providers’ counselling could have on changing male partners’ behaviours and attitudes towards IPV in the long-term, especially in a context in which social norms condoning IPV were pervasive.

Husband is counselled, but when he goes home he changes, he becomes evil. Most of them [men] change. He could be counselled very well, but when you go home … maybe the people he normally hangs out with and talks to can influence him, he changes for the worse, some initially they are good. [14, 2 children, on ARVs, married]

Both abused and non-abused women mentioned couple counselling on how to reduce partner blame, stigma and conflicts following disclosure as a way to reduced IPV and prevent separation. Providers could also help ‘normalize’ HIV and reduce stigma by providing education to couples.

They [providers] should always insist that couples must be tested together so that each is counselled and both will be contented with the counselling given so there will be no cause of quarrels between the couple. Because no one is willing to take responsibility for infecting the other with the virus, couples should be counselled and made to understand HIV virus can infect anyone and anyone can infect the other so no need for quarrels and separations. [05,1 child, on ARVs, separated]

They [health providers] should counsel people to avoid game blame. People should be made to understand that HIV virus is now common and if it has happened the matter [of blame and how is responsible for HIV transmission] should rest and they carry on with life. [09, 4 children, on ARVs, married]

Joint counselling prior to HIV testing could also be used by health providers to reduce partner blame. Others reported that the fear of partner violence could inhibit disclosure and thus providers could ask also women to bring their husband to the clinic for mediated disclosure.

[…] Personally I would choose not to tell my partner if I am found to be positive and I suspect he will be violent towards me. Or the woman can be told to go and ask the partner to accompany her back to the clinic [for disclosure]. [05, 1 child, on ARVs, separated]

A special mention was given to sero-discordant couples and the importance of counselling in order to protect women from abuse and HIV transmission, but also to preserve a marriage and prevent partner abandonment.

[…] after discovering my HIV status, we could have talked as a couple … […] and we could have lived a good life. We should have gone for counselling on how to live positively [as he was negative], but he decided to chase me away. […] They [providers] can attend, but it is difficult to explain to some people to understand, like me and him [my husband] we needed proper counselling and this could have saved our marriage or helped. [02, 2 children, on ARVs, in relationship]

Only a minority thought that health providers can only help medically and should not interfere in people's lives.

… even if she is beaten and she is back at her home how can the provider be of any help? If she is beaten, will she first go to the chief, to the police or to the provider? She will first go to the police obviously [Respondent is shouting almost] … […] and maybe you were just chasing each other around and you are not hurt, and then you run to the provider, what will the provider do? You see the provider will only treat you if you have been hurt. [07, 2 chizldren, on ARVs, separated]

I was told to go and tell him but he said that he doesn't want [to get tested] […] No [I do not think providers can help] because you can interfere in two people's affairs. [01, 2 children, on ARVs, separated]

## Discussion

Nearly a third of the respondents reported experiencing physical and/or emotional violence by their partners following sero-disclosure, suggesting that HIV status disclosure can be a period of heightened risk for partner stigma and abuse, and financial withdrawal, and thus should be handled with caution, as acknowledged elsewhere [9–11]. Women in our study reported relational triggers such as sero-discordance, asymmetrical access to HIV testing and disclosure, and requesting partner testing. Similar findings among African sero-discordant couples also report prevalent IPV if the male has negative or unknown HIV status [5,11], and higher rates of marriage and relationship dissolution [32,33].

In our study, sero-concordance was protective for emotional and verbal IPV once the partner knew his positive status and/or knew the woman knew his status. This finding aligns with another study among pregnant women in Zimbabwe that found that a decrease in severe violence was associated with women's partners knowing their own HIV status [34], implying that men who know their own status may enact less violence. This exploratory finding should be confirmed in future research.

Overall, our results show acceptance of the role of the health services in helping prevent and reduce anticipated fear of partner stigma and violence as barriers to HIV disclosure. However, given that our sample was existing users, probably with a relationship to staff already, it is important to note that non-users may see this differently.

Some of the approaches suggested by our respondents included couple counselling (especially for sero-discordant couples), separate counselling sessions for men, and facilitated disclosure. The women's narratives illustrate the importance of integrating discussions on IPV risks and fear of disclosure into HIV counselling and testing (HCT), helping women develop communication skills about how to disclose their status, and reducing fears about marital separation and break-up. These are important considerations in both individual and couples HIV testing and counselling (CHTC), also suggested in other studies [5,25]. Moreover, although women in our sample stated that they wanted health providers to assist with IPV, other studies report that very few actually pursue such assistance [35].

Women in our study also expressed the key preventive role of health services in reducing blame for HIV transmission and raising awareness on HIV as a chronic disease. However, several women, including most who experienced partner abuse post-disclosure, reported receiving no counselling on how to safely disclose HIV status. These missed opportunities diminish the potentially preventive role that providers can play in reducing IPV risks for WLWH, and can potentially endanger women’ safety. Women's fear of partner disclosure may be a warning sign of ongoing IPV or unsupportive relationships and thus providers should be able to recognize these signs and respond accordingly. Integration of IPV identification, care and counselling into SRH and HCT/HIV services for WLWH could be a way forward, though it will require adaptations and system changes, including the prioritization and mobilization of resources, staff training and strong referral networks [36,37].

Women's encouragement to disclose or to bring their partners for testing has been a common strategy for HIV prevention, one also adopted by the Kenyan National Guidelines for HCT [38]. There are some accounts of coerced disclosure from South Africa [23] and India [39]. However, the new WHO Consolidated Guidelines on HIV testing services recommend that couples and partner HCT should be voluntary; and that providers assess potential risks for IPV and support people's decisions not to test with their partners [40]. In cases in which violence is a risk, the necessary referral should be make, and alternative models of disclosure should be offered [12,18,41,42], such as facilitated disclosure [25]. Additionally, the alternative model of delaying disclosure or choosing not to disclose altogether for the purpose of safety should also be considered. There is also the recognition that an individualized plan for safe disclosure should be adopted because each case is different. Health providers should adapt pre- and post-test counselling accordingly [18].

As raised by some study respondents, targeting male partners is key, and couple counselling may have a greater effect on male behaviour change [43], and could be used to help couples start communication on HIV testing [44,45]. However, further research is needed on how to best leverage the potentially protective effect of CHTC, especially among sero-discordant couples [33,46]. Considering that only a small percentage of male partners come for testing, additional research is also needed to find strategies to involve men in HCT, such as having male providers and making SRH and HCT clinics more male friendly [21,28]. Furthermore, although male involvement in SRH services can have beneficial health outcomes for women (and infants), it should not prevent women from accessing SRH services if they are not accompanied by their spouses. Health providers should not pressure SRH women to disclose or to bring their spouses to SRH and HIV services. Doing so could have adverse effects on women, as seen elsewhere in Kenya [47].

### Limitations

Limitations of this study included the fact that interviews focused on experiences of IPV post-HIV infection; therefore, it is not possible to fully determine whether sero-disclosure is an additional trigger in already abusive relationships. Moreover, this study may not have captured those women who were unable to attend services due to IPV or other factors, and further research among WLWH not attending services should be conducted to address these women's needs. Nevertheless, study findings offer additional insight to inform future research on integration of IPV discussions into HIV testing, adding to research evidence.

## Conclusion

Building on previous research that focused on ANC clients, this study makes an original contribution to the field of HIV partner disclosure by exploring IPV risks among non-pregnant women accessing SRH services. We show that a range of violent reactions and stigma from partners is also experienced by this group. It also investigates non-pregnant women's acceptance of the role of health workers in recognizing and responding to IPV, but also in potentially contributing to negative consequences through their lack of careful attention to the risks of HIV status disclosure to spouses. The findings suggest that health providers should be more cautious when asking WLWH to bring their partners for testing, by offering alternative models to ensure safer disclosure to partners. The health sector can also play a preventive role by sensitizing all SRH providers to potential IPV risks following disclosure, ensuring women's decision to disclose is fully informed and voluntary, and helping reduce the culture of blame on women for HIV transmission.
